# The Future of Stem Cells and Their Derivates in the Treatment of Glaucoma. A Critical Point of View

**DOI:** 10.3390/ijms222011077

**Published:** 2021-10-14

**Authors:** Simona Delia Nicoară, Ioana Brie, Ancuța Jurj, Olga Sorițău

**Affiliations:** 1 Department of Ophthalmology, “Iuliu Hatieganu” University of Medicine and Pharmacy, 8 Victor Babeș Street, 400012 Cluj-Napoca, Romania; 2 Clinic of Ophthalmology, Emergency County Hospital, 3–5 Clinicilor Street, 40006 Cluj-Napoca, Romania; 3 “Ion Chiricuță” Institute of Oncology, Laboratory of Cell Biology and Radiobiology, 34–36 Republicii Street, 400010 Cluj-Napoca, Romania; ioanabrie@yahoo.com (I.B.); olgasoritau@yahoo.com (O.S.); 4 Research Center for Functional Genomics, Biomedicine and Translational Medicine, “Iuliu Hațieganu” University of Medicine and Pharmacy, 8 Victor Babeș Street, 400012 Cluj-Napoca, Romania; ancajurj15@gmail.com

**Keywords:** stem cells, exosomes, glaucoma, miRNA, neurodegeneration, neuronal regeneration

## Abstract

This review focuses on the clinical translation of preclinical studies, especially those that have used stem cells in the treatment of glaucoma, with an emphasis on optic nerve regeneration. The studies referred to in the review aim to treat optic nerve atrophy, while cell therapies targeting other sites in the eye, such as the trabecular meshwork, have not been addressed. Such complex and varied pathophysiological mechanisms that lead to glaucoma may explain the fact that although stem cells have a high capacity of neuronal regeneration, the treatments performed did not have the expected results and the promise offered by animal studies was not achieved. By analyzing the facts associated with failure, important lessons are to be learned: the type of stem cells that are used, the route of administration, the selection of patients eligible for these treatments, additional therapies that support stem cells transplantation and their mode of action, methods of avoiding the host’s immune response. Many of these problems could be solved using exosomes (EV), but also miRNA, which allows more targeted approaches with minimal side effects.

## 1. Introduction

Glaucoma, the most common of the optic neuropathies [1], is not a single entity, but includes a variety of diseases having in common the progressive loss of the retinal ganglion cells (RGCs) and the typical excavation of the optic nerve head (ONH) [2,3]. Glaucoma is one of the leading causes of irreversible loss of vision, currently affecting 60 million people worldwide, of which 8.4 million are blind [4]. It is estimated that this figure will rise to 111.8 million by 2040 [4]. Glaucoma is found in about 1–4% of all humans older than 45 years [3]. Tham et al. estimate a global glaucoma prevalence of 3.54% in the population aged between 40–80 years [4]. In classifying glaucoma, three main parameters are used: whether it is primary (idiopathic) or secondary (associated with other ocular or systemic conditions), the opening of the anterior chamber angle (open or closed) and the acute or chronic state. The most prevalent form in Europe and North America is primary open angle glaucoma (POAG), and in East Asia, primary angle closure glaucoma, accounting for at least 74% of all cases [3,5]. For POAG, prevalence in people over 70 years was estimated at 3% in Asian, 6% in white and 16% in African populations [2].

In humans, the optic nerve is formed by the axons of 1.2 million RGCs that receive their input from more than 100 million photoreceptor cells, via the intermediate cells (bipolar, horizontal and amacrine cells) [2]. The number of RGCs decreases with age, at a rate of approximately 0.5% per year, but within the time frame of a life span, this reduction has no consequence on vision [2]. RGCs axons leave the eye as the optic nerve through the lamina cribrosa, a connective tissue equipped with pores lined with glial support cells. This region is called ONH. The optic nerve becomes myelinated after having passed through the lamina cribrosa. The laminar region has high metabolic demands, as proved by the increased cyclooxygenase activity and the high density of sodium channels and mitochondria at the level of this unmyelinated portion of the ONH, as compared to the myelinated optic nerve [6]. This also explains the high vulnerability of the intralaminar axons of the optic nerve to metabolic stress [5]. After visual information is processed at the retinal level, the optic nerve conducts it to centers in the brain where it is further transformed: the lateral geniculate body (from which the visual information gets to the visual cortex), the superior colliculus and the suprachiasmatic nucleus. These centers are responsible for visual perception, eye movements and circadian rhythms, respectively [2].

## 2. Pathogenesis of Glaucoma

The main risk factor for glaucoma is raised intraocular pressure (IOP), which is regulated by aqueous humor (AH) physiology [2,3]. The ciliary body continuously secretes the AH into the posterior chamber of the eye, from where it is drained through the trabecular meshwork (TM) and the uveo-scleral pathway [3]. Raised IOP seems neither sufficient nor necessary for the development of POAG, but it is strongly associated with it [3]. Only one-third to half of the patients have elevated IOP in the initial stages of POAG [7]. There are patients who develop glaucomatous optic nerve damage in the presence of IOP within the normal range (12–22 mmHg), in the framework of normal tension glaucoma (NTG). On the other hand, some individuals with raised IOP do not develop glaucomatous optic neuropathy, or the disease still progresses in some patients when lowering IOP in NTG [8]. Traditionally, the pathophysiological viewpoints of glaucoma that are based only on raised IOP include two main theories: mechanic and vascular. However, recent studies regarding the development of NTG highlighted some IOP-independent risk factors besides the vascular ones, such as trans-laminar pressure difference (TLPD), immune disorders, neuroinflammation, genetic factors and myopia-related biomechanical factors [8]. 

### 2.1. Mechanic Theory

According to the mechanic theory, the induction of the glaucomatous optic neuropathy is a direct consequence of RGCs axons’ compression. Increased IOP compresses the capillaries at the level of the ONH, resulting in impaired blood flow, obstruction of the axoplasmic flow and ultimately, chronic ischemic injury of the optic nerve. Histological studies proved alterations in the structure of the lamina cribrosa in the early stages of the disease, consisting of compaction and fusion of its layers and loss of RCGs axons, glial cells and capillary vessels [9]. Subsequently, NTFs (neurotrophic factors) cannot reach RGCs soma and they accumulate at the level of the lamina cribrosa. Experimental models have shown that raised IOP also damages RGCs mitochondria [2]. The deprivation of growth factors and mitochondrial damage shift the expression of RGCs genes from cell support to pre-apoptotic pathways [2]. 

In vitro studies have proven that raised IOP activates the retinal glial cells (Müller cells, astrocytes, and microglia) with the subsequent release of neurotoxic substances, such as nitric oxide (NO) and tumor necrosis factor α (TNF-α), resulting in further damage of RGCs. ONH astrocytes secrete matrix metalloproteinases (MMP) and extracellular matrix (ECM) molecules and increase the synthesis of transforming growth factor-β2 (TGF-β2) in the region of the lamina cribrosa, inducing the alteration of ECM proteins [10]. These processes might explain the in vivo shaping of the lamina cribrosa and initiate the structural (axon atrophy and degeneration) and biochemical changes of the axon environment [2]. 

This theory cannot explain NTG or the fact that some patients with glaucoma continue to lose vision despite controlled IOP by medication or surgery. Mechanisms that are independent of IOP could be involved in the induction of glaucomatous degeneration. Glaucoma has similarities with other neurodegenerative conditions such as Alzheimer’s and Parkinson’s diseases, in which selective loss of neuron populations and transsynaptic degeneration occur, with spreading of the disease from the injured neurons to the connected ones. It seems that NTG induces damages that are not limited to the eye, but extend to the entire visual pathway, as well as to some nonvisual pathways in the brain [8]. It was observed that the patients affected by ocular hypertension, glaucoma, demyelinating optic neuritis and Alzheimer’s disease presented a reduction of retinal nerve fiber layer (RNFL) thickness as evaluated by OCT, which was correlated with the electrophysiological responses of the retina (assessed functionally by Pattern electroretinography (ERG) recordings) [11,12,13]. 

### 2.2. Vascular Theory

The vascular theory of glaucoma proposes a mechanism independent of IOP, arguing that RGCs loss is the consequence of insufficient ocular blood flow induced by vasospasm and hypoxia. One argument in support of this theory is the association of glaucoma with vascular diseases, such as hypertension and diabetes, suggesting the possible involvement of genetic or familial factors, especially in POAG [14,15]. Generalized shrinking of the retinal vessels as a typical aspect of advanced glaucoma damage was observed in a study on retinal microcirculation, which consisted of the morphometric analysis of optic disc color photographs in patients with POAG [16]. Endotheliopathies are other entities that can be involved in the pathogenesis of glaucoma, following the identification of a relationship between reduced vascular endothelial progenitor cells and reduced flow-mediated dilation (FMD) [17]. FMD in the brachial artery is an index of vasomotor function that is quantified non-invasively by evaluating the vascular response to NO and thus assessing the endothelial function. Liu et al. (2016) observed a correlation between baseline FMD and Humphrey visual field (VF) progression in the inferior peripheral field in patients with NTG and POAG, suggesting that peripheral vascular endothelial dysfunction may be related to glaucoma progression [18]. Diseases with compromised vascular endothelial cell function, such as the Raynaud phenomenon, ischemic vascular diseases and migraine headache, have a higher prevalence in patients with NTG, suggesting the involvement of a vascular mechanism in the pathogenesis of glaucoma [19]. “Endothelial dysfunction” is defined by endothelium-dependent vasodilation and “endothelial activation” is characterized by a proinflammatory and proliferative status [20]. Endothelium has a primary role in the regulation of blood flow through responses to vasoactive agents and hormones and by releasing vasodilator substances such as NO or the vasoconstrictor endothelin-1 (ET-1). Imbalance between NO and ET-1 leads to ischemia or vascular dysregulation [21]. The direct relationship between the reduction of blood flow at the level of ONH and the damage of RGCs in glaucoma could not be proved, although experimental studies on animals might support this hypothesis [2]. Endogenous vasoconstrictors, such as ET-1, might cause ischaemic damage of RGCs at the ONH. Studies proved that patients with glaucoma have an increased level of ET-1 in the aqueous, and that they react to cold-induced stress by increased plasma levels of ET-1 [22,23] which were not observed in non-glaucomatous individuals [24]. In addition to its vasoconstrictive effect, ET-1 also activates astrocytes at the ONH [25,26] with subsequent neurotoxic and matrix shaping effects. In some individuals with POAG, there is vascular dysregulation that can cause chronic impairment of ONH blood flow. Primary vascular dysregulation includes Raynaud syndrome and migraine, while secondary dysregulation includes all conditions characterized by increased levels of ET- 1, such as rheumatoid arthritis and systemic lupus erythematosus. Primary vascular dysregulation is a risk factor for some forms of glaucoma because the autoregulation of ocular blood flow is disturbed, leading to oxidative stress at the level of RGCs. Secondary vascular dysregulation does not seem to be a risk factor for glaucoma, as blood flow is reduced globally and vasoconstrictive peptides (such as ET- 1) are paracrine factors with raised systemic levels that are not necessarily dangerous for the eye [2].

### 2.3. Neuroinflammation

Glaucoma-related stimuli (optic nerve transection, ocular hypertension, excitotoxicity) induce glial cell activation with consecutive secretion of proinflammatory mediators that ultimately affect neuronal survival [27]. From this perspective, neuroinflammation is another important mechanism involved in the pathogenesis of glaucoma, possibly related to mechanical and vascular mechanisms. The connection between neuroinflammation and glaucoma has been proved in animal models, but there are also clinical data supporting it. The analogies with neurodegenerative diseases that were recently revealed to be correlated with inflammatory responses (amyotrophic lateral sclerosis, Alzheimer’s, Parkinson’s, Huntington’s disease, and frontotemporal dementia) suggest that similar cellular players, molecular mechanisms, genes and the microbiome are involved in glaucoma-related neurodegeneration [28,29,30]. Common features of these neurodegenerative diseases are strongly age-related incidence, and similar mechanisms of cell injury (oxidative stress, mitochondrial dysfunction, alterations in the ubiquitin-proteasome system, glutamate excitotoxicity, glial activation and inflammation, deposition of protein aggregates in specific anatomical areas) with the result of neuronal death [31]. The central nervous system (CNS), and particularly the brain, retina and optic nerve, are immune privileged tissues, where the interaction with the immune system is restricted by the brain–blood/retinal–blood barrier [32]. Glaucoma related neuroinflammation can be located in different compartments of the eye (retina, ONH), optic tract and brain (superior colliculus and lateral geniculate), but also in the blood, bone marrow or other tissues [33]. The main cells involved in the inflammatory responses within the retina are microglia and macroglia (Müller cells and astroglia). Infiltration of leukocytes into the ONH and retina may be key events in glaucoma. These cells offer metabolic support to neurons, neuroprotection and regulate the synaptic activity. Microglial cells are CNS-resident innate immune cells, migratory blood monocytes to the CNS that retain monocyte-specific antigens CD11b/c, complement peptide C3a receptor 1 (C3aR1) and chemokine receptor (CX3CR1) [28,34]. The activation of microglia is an early event in the retina and optic nerve during glaucoma. Mature microglial cells are activated by damage-associated molecular patterns (DAMPs) and by heat shock proteins (HSPs) produced by RGCs when IOP is elevated. Activated microglial cells interact with astroglia, these two cell types being the most important actors in modulating the inflammatory immune response in the retina and optic nerve. Depending on this interaction via signaling proteins (such as complement factors, TNF-α) and their receptors (Interleukin-1β (IL-1β), IL-6) [27], astroglia are differentiated into A1 astrocytes with neurotoxic effects or A2 astrocytes with neuroprotective properties. The neuroinflammatory process also induces the differentiation of microglia into M1 (proinflammatory) or M2 (anti-inflammatory) activated macrophages [28]. The interplay between A1/A2 astrocytes and M1/M2 microglia will determine the extent of neuronal damages, i.e., RGCs death. Broadly, RGCs death is the result of two main pathways: impairing RGCs survival and initiating RGCs apoptosis.

### 2.4. RGCs Maintenance and Survival

In the normal eye, RGCs are continuously supported by NTFs from the brain via retrograde axonal transport and from the glial Müller cells. In glaucoma, the axonal transport is disrupted, preventing the brain-derived NTFs to reach RGCs [35]. ONH is the area which is most susceptible to the damage caused by raised IOP, more specifically, to the IOP gradient across the lamina cribrosa. The retrobulbar optic nerve is surrounded by a cuff of cerebrospinal fluid (CSF). In several individuals with glaucomatous VF defects, an extremely low CSF pressure was measured, resulting in a high TLPD [36]. According to the “neurotrophin hypothesis”, the disruption of the axonal transport by the mechanical damage at the ONH leads to RGCs NTFs starvation which impairs RGCs survival and promotes their apoptosis. This hypothesis is supported by evidence from experimental glaucoma [37]. However, other findings suggest that there is a compensatory retinal response to injury, suggesting the involvement of other growth factors [38].

### 2.5. RGCs Apoptosis

The major neurotransmitter in the retina that mediates the transmission from the photoreceptor to bipolar cells and further on to RGCs is glutamate. In several neurodegenerative conditions, such as Alzheimer’s, Parkinson’s and Huntington’s disease, lateral amyotrophic sclerosis and cell death following stroke, it has been proved that excessive levels of glutamate are toxic to cells and initiate apoptosis [39]. However, the direct link between high glutamate levels and glaucomatous optic nerve damage in humans was not established. Moreover, a study performed on humans proved similar glutamate levels in the vitreous of glaucomatous versus control eyes [40]. One possible explanation is that the retina has a glutamate clearance mechanism that reduces its in situ toxicity, as proved by in vitro retinal explants from glaucomatous rat eyes [41]. In a primate model of glaucoma, the glutamate levels were not higher in the vitreous of glaucomatous versus non-glaucomatous eyes and there was no correlation between the glutamate levels and the degree of RGCs loss [42].

## 3. Clinical Presentation

When IOP is too high for the health of the optic nerve, characteristic deformation of the ONH results, referred to as excavation or cupping [43]. Because the progression of VF defect is slow and painless, glaucoma is often diagnosed after a substantial percentage of RGCs have been damaged [6].

## 4. Therapeutic Strategies

Since raised IOP is the main trigger for glaucomatous optic nerve damage, most available therapeutic agents aim to drive its reduction [43]. IOP reduction by drugs, laser or surgery is the only clinically available and approved glaucoma treatment [3,35]. The treatment involves the daily administration of eye drops, and adherence to it is often unsatisfactory [43]. Drug classes are: β-blockers, prostaglandin analogues, topical or systemic carbonic anhydrase inhibitors, α_2_-adrenergic receptor agonists and parasympathomimetics. These drugs decrease IOP either by reducing aqueous production by the ciliary body epithelium, or by increasing its flow via TM or uveo-scleral pathway. Carbonic anhydrase inhibitors also improve blood flow at the ONH; brimonidine, betaxolol and latanoprost have also a neuroprotective effect [43]. If glaucoma continues to progress despite a combination of pharmacologic treatments, laser and surgical methods are applied to enhance aqueous flow or reduce its production [43]. In closed angle glaucoma, the iris tissue blocks the access of AH at the TM, resulting in acute or chronic elevation of IOP [3]. Surgery aims to unblock the flow and thus relieve the pressure [3]. In some cases, the need for chronic medical therapy may remain following surgery [3]. Results of surgery in glaucoma are limited by complications related to wound healing which worsen the outcome. There is no standardization in defining success and the number of clinical trials assessing success of glaucoma surgery is limited [43]. The approaches to lower IOP have proved to be efficient in delaying glaucomatous neurodegeneration and prolonging vision, but they are sometimes transitory in nature [1]. Fluctuations in IOP are well-known in glaucoma, limiting the effects of various therapeutic approaches [1]. In some individuals, despite IOP normalization, glaucoma continues to progress, because neural degeneration and apoptosis proceed, even if the initial insult was resolved. Therefore, the need to develop additional neuroprotective strategies seems logical. However, neuroprotection alone cannot solve the problem without correcting the initial issue [2]. The strategy to develop new approaches in glaucoma management includes three main directions of action: the development of new drugs to lower IOP or improve ONH perfusion, the administration of agents able to rescue damaged RGCs (neuroprotection) and the long-term goal to regenerate the optic nerve.

### 4.1. Optic Nerve Regeneration

Clinical studies and glaucoma patient trials with stem cells transplantation were preceded by numerous pre-clinical studies on the two main lines of bringing stem cell therapy to optic nerve restoration for glaucoma. The first direction was to understand the molecular pathways and mechanisms that determine stem cells to differentiate into specialized neurons and RGCs. The second direction was to look after the effects of stem cell transplantation and their integration within the eye tissues in pre-clinical models.

Until 1996, it was believed that the optic nerve axon was impossible to regenerate. Berry et al. (1996, 1999) discovered that implanting a peripheral nerve graft into the vitreous body of the eye with optic nerve crush determined RGCs to regenerate axons at least 3–4 mm into the distal segment [44,45]. Further studies in animal models used intravitreal administration of neurotrophic growth factors (neurotrophins), which are acquired by retrograde axoplasmic transport: brain-derived neurotrophic factors (BDNF), nerve growth factors (NGF), neurotrophin-3 (NT-3) and neurotrophin-4 (NT-4), neurokines (ciliary neurotrophic factor—CNTF). A delay of acute RGCs death has been shown, but the results were generally disappointing. One possible explanation is the rapid clearance of the NTFs [46]. It seems to be difficult to translate pre-clinical results to the clinical practice. Many neuroprotective drug candidates that have proven effective in animal models have failed clinical trials. Two examples are represented by memantine, a non-competitive *N*-methyl-d-aspartate (NMDA) subtype of glutamate receptor antagonist, already in use in the treatment of Alzheimer’s disease and brimonidine, a selective α-adrenergic receptor agonist. 

The animal models are limited by their incapacity to mimic the heterogeneity of human glaucoma and the presence of comorbidities. Furthermore, in humans the therapeutic interventions are made in a more advanced stage of the disease [47].

**Figure 1:**
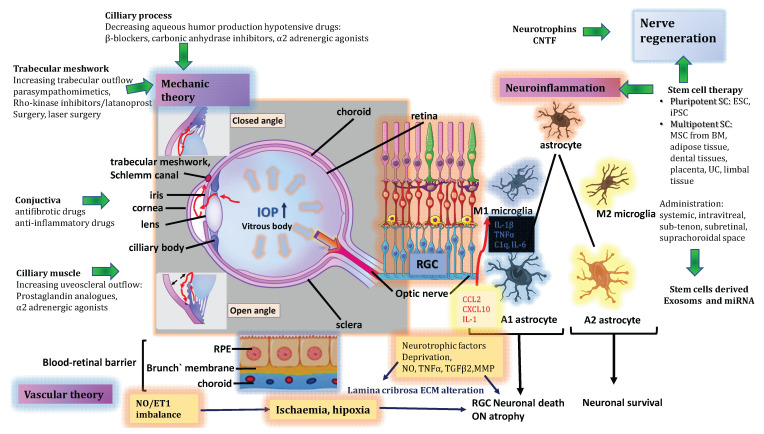
The main pathophysiological mechanisms in glaucoma and the mode of action of existing therapies in the clinical practice.

The main pathophysiological mechanisms in glaucoma and the mode of action of existing therapies in the clinical practice are illustrated in Figure 1. 

### 4.2. Stem Cell Therapies

Uncontrolled glaucoma leads to blindness, as it is associated with irreversible RGCs loss. In these patients, the only hope to restore vision is optic nerve regeneration. However, this direction of action is full of difficulties. First, a source of stem cells capable of differentiating into RGCs needs to be identified. These cells have to be transplanted in the eye and integrated into the retina, in an environment which should be permissive to axon re-growth. In the CNS, glial scars prevent neural repair. In glaucoma, the biochemical microenvironment is altered by activated astrocytes at the lamina cribrosa and Müller cells in the retina [48]. These changes need to be addressed to promote axon growth through the ONH. Finally, after having been transplanted and successfully integrated, the cells need to form synapses with retinal interneurons and their corresponding neurons in the brain, in order to preserve the retinotopic organization of the visual pathway.

Stem cells have come to the attention of researchers due to certain features that are advantageous for glaucoma therapy: (1) the ability to differentiate or to be reprogrammed into many types of cells, with the possibility of selective cell replacement of RGCs or other specialized cells within the eye; (2) the neuroprotective and immunomodulatory properties of certain types of stem cells, such as mesenchymal stem cells (MSCs) [49]; (3) the presumable low immunogenicity, especially for pluripotent stem cells; (4) the bioactivity of factors and molecules secreted by stem cells (the “secretome”), with roles in injury repair and immunomodulation, with proven therapeutic benefits rather than the integration of stem cells into the host tissue; this prerequisite can be achieved by using extracellular vesicles (EV) or miRNA [50]; (5) the possibility of using transplanted stem cells as intraocular delivery devices for the release of neurotrophic agents, growth factors, survival/anti-apoptotic factors with a prolonged and localized effect [49].

Based on encouraging glaucoma preclinical studies using pluripotent stem cells represented by embryonic stem cells (ESCs), induced pluripotent stem cells (iPSCs) and adult stem cells derived from different adult tissues, those cells were proposed for translation into human clinical research and applications. The advantage of pluripotent stem cells is their increased potential to differentiate into all somatic cell types and the possibility to be maintained in an undifferentiated state for a prolonged period in culture. iPSCs, described by Takahasi et al. in 2007 [51], are generated from somatic adult tissue by genetic reprogramming using defined transcription factors. Producing iPSCs from the patient’s own cells can prevent the immunological host response or reduce the immunosuppression therapy post-transplantation. A common problem regarding ESCs and iPSCs is genetic and epigenetic instability with subsequent development of teratomas and in vivo immunogenicity [52]. Another drawback of iPSCs is the more laborious cultivation technique in contrast with adult stem cells. MSCs are self-renewing multipotent post-natal cells [53] found in fetal tissues, placenta, the umbilical cord and adult tissue (bone marrow, adipose tissue, dental tissues, peripheral blood, skin, limbal tissue) [54]. Some important features recommend MSCs for transplantation: (1) they are capable of modulating the host immune system and other systems, with anti-inflammatory properties; (2) following transplantation, they are transient cells (days-2 weeks); (3) express homing receptors that enable MSCs to migrate to sites of damage and inflammation; (4) production of bioactive molecules: growth factors, chemokines, cytokines such as epidermal growth factor (EGF), vascular endothelial growth factor (VEGF)-A, fibroblast growth factor (FGF), platelet-derived growth factor (PDGF)-AB, hepatocyte growth factor (HGF), transforming growth factor (TGF)-b1, TNF-α, stromal cell-derived factor (SDF)-1α, IL-6, IL-8, insulin growth factor (IGF-1) with trophic and survival roles; (5) their immunogenicity is low, due to the fact that MSCs does not express major histocompatibility complex (MHC) class II molecules (HLA-DR), the costimulatory molecules (CD40, CD80, CD86) and hematopoietic markers CD45, CD34, CD14, CD11, CD19, and CD18 [55,56]. One disadvantage of MSCs is the possible low engraftment due to their short-life span after injection. Once differentiated, MSCs may have no therapeutic effect and display increased immunogenicity due to MHC-I and MHC-II expression. In systemic transplantation, many of them are trapped in the lungs. The risk associated with tumorigenesis after stem cell transplantation is not excluded and is widely discussed in the literature [56].

Miotti et al. 2021 performed an extensive overview of stem cells-based therapies applied to almost all parts of the eye in the past 20 years, and they identified more than 2000 studies [57]. The interest in cell transplantation for ophthalmic diseases is supported by certain features of the eye: a small number of cells is required, it is surgically accessible, and the grafts can be easily evaluated and visualized [57]. Although there is a huge interest in the topic of stem cell transplantation in glaucoma, demonstrated by the explosion of studies and reviews over the last decade (especially during the last 2–3 years), it is not yet possible to state with certainty the safety of this therapeutic approach in optic nerve regeneration. The major risks in patient trials with cell therapies are host graft disease, infection, inflammation, and more severe vision loss. Kuriyan et al. 2017 [58], drew attention by publishing a study in New England Journal on the situation of three patients with AMD in whom severe bilateral visual loss developed after they had received bilateral intravitreal injections of autologous adipose tissue-derived “stem cells” at a stem-cell clinic, which was the study site for a trial (NCT02024269) in the United States. Two other authors reported retinal detachments in patients with AMD after having received bilateral intravitreal injections of “stem cells” and were published in 2016 and 2017, respectively [59,60]. Such unfortunate cases are a warning about the importance of proper testing in pre-clinical models before moving towards human trials.

### 4.3. Principles of Clinical Protocol Development for Stem Cell Therapies

A well-controlled clinical trial with safety results that will then be accepted by academic communities and applied by clinicians is a randomized, masked clinical trial. Such a study can be expensive and lengthy, and cannot be applied in all situations. Good manufacturing practice (GMP) is a principle in leading and conducting these trials.

General steps to follow in the design of glaucoma clinical trials are: (1) analysis of the results of later phase clinical trials based on early phase clinical trials and animal studies; (2) defining parameters for glaucoma assessment, such as IOP and its measurement method; (3) defining the criteria for patient inclusion; (4) evaluation of therapeutic effects based on clinical and paraclinical data; and (5) planning clinical trials according to the number of subjects, dropout rates, estimated serious adverse events and protocol violations [61]. Specific steps in stem cell therapies trials are: (1) the selection of cell type: autologous (same or fellow eye, other tissues of the patient) or allogenic (from a donor). The autologous stem cells transplantation has the advantage of not requiring immunosuppressive therapy, thus eliminating the risk of immune reactions. The availability of cells is another aspect to consider: iPSC are safer, but the costs of obtaining and cultivating them are higher. MSCs from adipose tissue or umbilical cord are more available and cost effective. Another aspect is whether to use undifferentiated or pre-differentiated cells. Pre-differentiated cells require a well-characterized cell population with the appropriate identity at a specific stage of differentiation. The cells must be characterized phenotypically, genotypically and functionally. (2) The route of delivery is a particularly important issue in ophthalmology. (3) The necessity of NTFs supplementation depends on the type of the proposed regeneration (4). The prediction of host environmental modification depends on the balance between the benefits of therapy and its adverse reactions [49,57,62]. 

### 4.4. Clinical Trials Using Stem Cells in Glaucoma

So far, only a few cell therapies have been approved for eye disease to be transposed into clinical practice for some inherited retinal dystrophies and age-related macular degeneration (AMD), with controversial results. A clinical trial using Palucorcel (CNTO-2476), a human umbilical cord tissue derived cell compound injected in the subretinal space, reported a high incidence of retinal perforation and retinal detachment in patients with geographic atrophy (GA) secondary to AMD [63]. Other phase I/II trials have evaluated the safety of RPE transplantation in patients with advanced dry AMD-related GA (NCT01344993) and Stargardt disease (NCT01345006). RPE derived from human embryonic stem cells (MA09-hRPE) were delivered to the subretinal space after standard vitrectomy. The patients responded favorably to cell therapy with improvement in best corrected visual acuity (BCVA) and no evidence of adverse proliferation, rejection or serious ocular or systemic safety concerns, with the adverse events deemed to be associated to the surgery and immunosuppression [64]. A phase I (*n* = 10; NCT00063765) and II trial (*n* = 51; NCT00447954) were approved for glaucoma, to evaluate the safety of CNTF and RGC neuroprotection, delivered over a 6-month period by encapsulated cells implanted intravitreally. The treatment was safe, but with no significant improvement in the evolution of the disease [65,66]. Another clinical trial (ClinicalTrials.gov, NCT02330978, (accessed on 1 May 2015) which was conducted from 2015 in two patients with POAG in advanced stage of optic neuropathy, used a single intravitreal injection of autologous bone marrow-derived MSCs. ERG findings showed no response after treatment. One patient showed retinal detachment at day 15 with proliferative vitreoretinopathy. The other patient had stable ERG responses throughout the study period [67]. Another trial for the therapy of early-stage glaucoma started in 2014 in the Russian Federation (NCT02144103- 16 patients enrolled), using autologous MSCs from adipose tissue. The results have not yet been reported [68]. 

After a search of the ClinicalTrials.gov database, we summarized in Table 1 the clinical trials investigating stem cell therapies in glaucoma and optic nerve atrophy that are registered at the National Institute of Health. Unfortunately, there are few registered clinical trials, many of them having not reported any results, and enrolling a small number of patients, except for SCOT1 and 2 clinical trials, that used autologous BM-MSCs for the treatment of several diseases of the retina and optic nerve, in a lesser extent in glaucoma patients. Weiss et al. elaborated several publications from 2015 to 2021, with the results of SCOT 1-2 trial with stem cell therapy in autoimmune optic neuropathy (1 case) [69], sequential non-arteritic ischemic optic neuropathy (NAION) (10 cases) [70], dominant optic atrophy (6 patients) [71], AMD (32 eyes) [72], Leber’s hereditary optic neuropathy (5 patients) [73]. Treatment approaches included a combination of retrobulbar, sub-tenon, intravitreal, intra-optic nerve, subretinal, and intravenous injection of autologous BM-MSCs according to the nature of the disease, the degree of visual loss and treatment-related risk factors. The authors reported a relatively high percentage of patients having achieved meaningful visual improvements. The procedures were considered to be safe, and no serious complications were observed. However, it is worth noting that patients with glaucoma-induced optic nerve atrophy were not included in the reports of SCOT1-2 trials.

Between 2015 and 2019, Limoli et al. conducted a clinical trial that was registered at the Low Vision Center in Milan [75]. They injected adipose tissue derived MSCs as suprachoroidal autograft, using the Limoli retinal restoration technique (LRRT), in 35 eyes with glaucomatous optic neuropathy (GON). This restoration technique consists of the administration in the suprachoroidal space (SCS) of an autologous cellular triad: adipose stromal cells (ASCs), adipose-derived stem cells (ADSCs) contained in the stromal vascular fraction (SVF) of adipose tissue and platelets (PLTs) recovered from the platelet-rich plasma (PRP). Each patient was subjected to a complete ocular examination: BCVA for far and near vision, sensitivity by microperimetry and spectral domain-optical coherence tomography (SD-OCT). At six months, in 21% of the patients with GON treated with LRRT, the disease had not progressed, and in 79% there was a significant increase in visual performance, as compared to the control group (matched GON patients according to the sensitivity alteration measured by microperimetry) [75]. This study stands out by its design, with noticeably clear and realistic inclusion and exclusion criteria (e.g., patients with systemic diseases such as multiple sclerosis, epilepsy, vasculitis, Parkinson’s disease, renal and hepatic diseases, malignant neoplasms, and decompensated diabetes mellitus), characterization by flowcytometry of isolated stem cells and platelets and clinical and paraclinical follow-up. The LRRT technique seems to be beneficial and safe by using SCS. Other routes, such as the intravitreal and subretinal ones, are reported to develop severe side effects. The suprachoroidal area of only 25 mm^2^ is a space between the sclera and choroid that circumferentially spans the entire posterior segment of the eye, and it has been shown to be a natural drug storage and an immune-protected region. From this space, growth factors secreted by stem cells can pass and reach the retina without producing immune reactions [75,76,77]. There are several methods to assess the SCS: surgical procedures, standard hypodermic needle, hollow microneedles. The surgical approach consists of a sclerotomy through which a catheter or canula is inserted to reach the posterior target. The method carries the advantage of good visualization since the catheter is guided with a flashing diode, but the disadvantage of invasiveness. The use of a standard hypodermic needle is less invasive, but since there is no visualization, great skill is required in order to achieve precise injection. The most promising method for SCS administration is the use of microneedles whose length are designed to fit the approximate thickness of the sclera and have important advantages: minimally invasive, very good safety profile, simple to use, less painful, inexpensive [78]. In addition to stem cells, PRP was also used by Limoli et al., which is also a source of growth factors and other bioactive molecules that are necessary for wound healing. PRP represents a relatively new approach in regenerative medicine that is obtained from patient’s own blood and contains growth factors (EGF, IGF1, HGF, TGFβ, VEGF, PDGF), chemokines (CCL5, CCL3, CXCL8), and clotting factors (factor V, factor IX, antithrombin, factor S, and their inhibitors) [79]. A phase 1/2 study that used the suprachoroidal route for patients with optic atrophy for implantation of umbilical cord-derived MSCs (UC-MSCs) (29 eyes) [80] or adipose tissue-derived MSCs (4 eyes) [76] has documented that this route of administration is safe and showed that stem cell therapies have led to improvement of BCVA and VF that were sustained by multifocal ERG recordings. 

### 4.5. Stem Cell Derived Exosomes

Most of the effects obtained from stem cell transplantation are mediated by their derivatives, the exosomes. Starting from this idea, it is understandable that exosomes could be a viable alternative to cell therapies, thus avoiding some worrying aspects such as immunogenicity. Exosomes were discovered in late 1980s and were initially considered as a manner through which the cells dispose of debris. Since then, exosomes gained attention in several fields, due to the diverse molecular species engulfed in their structure, ranging from nucleic acids, proteins, lipids, metabolites and others. Because of the wide variety of molecular species present in the structure of exosomes, they have the potential of being important biomarkers for diagnosis and prognosis in different diseases. Exosomes are small entities with a size ranging between 40 and 150 nanometers in diameter [81]. They are generated through the inward budding of late endosomes, thus producing multivesicular bodies (MVBs). Further, MVBs fuse with the cell membrane, releasing the exosomes in the extracellular space [82]. During biogenesis, exosomes are loaded with several bioactive molecules such as nucleic acids (miRNAs, lncRNAs, DNA), lipids, metabolites and specific proteins from the donor cells, which can be delivered to a target cell. According to their cell origin, exosomes present various loads, thus exhibiting different biological functions. Because of this, exosomes have an important role in intercellular communication and discharge of excess molecules [83]. These entities are a novel mode of intercellular communication and are able to modulate a wide range of biological processes in different malignant diseases [84] as cancer prognostic markers and anticancer drug-carriers [85]. 

**Figure 2:**
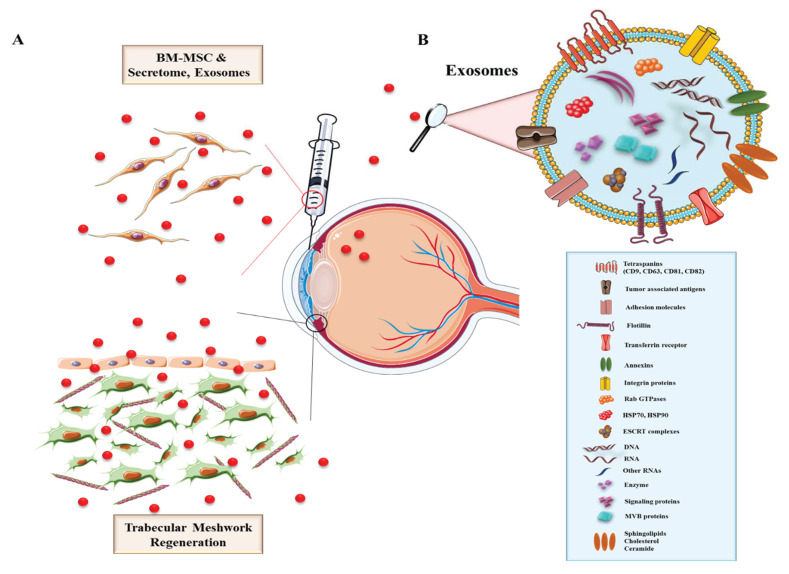
The uses of MSCs for ocular protection and regeneration. (**A**) MSCs-derived exosomes and neurotropic factors induce the regeneration of the trabecular meshwork. (**B**) The main components of exosomes, such as lipid bilayer (cholesterol, sphingolipids, ceramide), intracapsular proteins (MVB proteins, signaling proteins, HSP70, HSP90, Rab GTPases), various proteins (annexins, flotillin, tetraspanins, integrins), enzymes, nucleic acids (DNA, RNA, miRNAs), tumor associated antigens, ESCRT (endosomal sorting complex required for transport).

In addition to the cargo specific for the donor cell, exosomes also have markers that are relatively constant between different donor cells, generally termed as “exosomal marker proteins”. These markers generally reflect the mechanism of exosome formation like ESCRT (endosomal complexes required for transport) proteins, alongside its accessory proteins (Alix, HSC70, HSP90β, TSG101) [86]. More than that, exosomes contain certain markers that are specific to endosomes (CD9, CD63, CD81, CD82), membrane proteins needed for fusion and cell transport (annexins, flotillin, GTPase) and phospholipases [87]. Conversely, other markers are rarely if at all present in exosomes. For example, proteins associated with mitochondria or with the nuclear membrane are not seen in exosomes, while proteins associated with the endoplasmic reticulum and the Golgi apparatus are seen in low levels in exosomes [88] (Figure 2).

It has been shown that MSCs produce more exosomes compared to other cells [89]. MSC-derived exosomes express surface proteins common for all exosomes, but also adhesion molecules which are specific for MSC membranes, like CD29, CD44 and CD73 [90]. According to their small size, MSC-derived exosomes are excellent vectors for drugs or DNA constructs, being used as potential resources for cell and gene therapy [91]. 

There is evidence for the implication of exosomes in different diseases, like glaucoma. Pan et al. [92] demonstrated the implication of exosomes isolated from umbilical cord MSCs in a rat model of optic nerve squeezing. Thus, it was observed that exosomes can stimulate the survival of RGCs, but do not promote axonal regeneration [92]. In a rat optic nerve crush (ONC) model, it was observed that exosomes secreted by the bone marrow MSCs significantly stimulated the survival and axonal regeneration of retinal ganglion cells through the transfer of miR-17-92, miR-146 and miR-21. The expression of a major inhibitor of retinal ganglion cell axonal growth, *PTEN*, is inhibited by MSC-derived exosomes through the loaded miR-17-92 and miR-21, while *EGFR* expression is reduced in a similar manner, but through the load of miR-146a. The inhibition of *EGFR* is important as, normally, it is involved in axonal suppression. The neuroprotective effect of bone marrow MSC-derived exosomes has been demonstrated in a DBA/2J mouse model of glaucoma, inducing the preservation of RGCs and protection of the axons [93]. Exosomes isolated from human embryonic stem cells (ES-MSCs) promote neuroprotection and functional preservation of RGCs in ONC mice. In this regard, ES-MSC can be used as a potential candidate for an adjunctive therapy of RGCs degeneration [94]. Moreover, MSC-derived exosomes, containing miR-21, were shown to have a protective influence on photoreceptor cells when exposed to N-methyl-N-nitrosourea [95]. MSC-derived exosomes also have an important role in immune modulation through a variety of actions, including the effect on macrophage polarization, by skewing them to an M2 polarized phenotype [96]. As MSCs have a direct action on different immune cells, it is not surprising that they are also involved in cytokine signaling, by modulating an exaggerated inflammatory cytokine response [97]. Through the injection of MSC-derived exosomes, it was observed that the expression level of *MCP1/CCL2* mRNA was inhibited in primarily cultured retina, promoting the recovery of retinal laser injury. This is important, as it has been demonstrated that MSCs and MSC-derived exosomes have beneficial effects on retinal laser injury repair [98]. Interestingly, MSCs can be primed by TNFα to exhibit a higher neuroprotective role, showing the role of these cells not only in general immunomodulation, but also in a negative feed-back loop needed to inhibit an already initiated inflammation [99]. Thus, it can be seen that MSC-derived exosomes have a general protective effect on all retinal layers, and a general immunomodulatory effect, which argues their use in ophthalmology. The first trials began in 2014 and most of them were dedicated mainly to oncologic, autoimmune and neurological diseases. To date, no clinical study has been initiated in glaucoma patients. As the pathophysiological mechanisms of glaucoma are elucidated, and technologies for obtaining exosomes will be developed and standardized, it is likely that this will be one of the future therapies in glaucoma.

### 4.6. miRNAs in Glaucoma

It is clearly demonstrated that MSCs are able to home to the damaged tissues and differentiate into many cell types. However, only a small fraction of the transplanted MSCs can engraft in the host tissues. In recent years, the literature stands for the observation that the paracrine soluble factors secreted by MSCs are responsible for the reparatory effects in damaged tissues through a mechanism which is mediated by EVs [100] such as exosomes. More and more evidence has revealed that EVs mediate the effects of originating cells via miRNA delivery.

miRNAs are small (19–24 nucleotide) non-coding single-stranded RNAs which modulate the expression of up to 30% of all mammalian protein-encoding genes [101], acting in the post-transcriptional regulation of genes expression by base-pairing with their target messenger RNA (mRNA). miRNAs are present in biological fluids and are of great promise as diagnostic and predictive biomarkers in various diseases [102,103]. 

Glaucoma, the most common cause of irreversible blindness worldwide, is difficult to be diagnosed early in its evolution, causing irreversible harm before any discernable vision loss. Therefore, there is a great need for molecular biomarkers for its early detection, thus making the disease more amenable to treatment. Altered expression of specific miRNAs was identified in neurodegenerative diseases (Alzheimer’s, Parkinson’s) and was also observed during glaucoma development [104,105,106]. Several studies found miRNAs involved in the pathophysiology of glaucoma. Drewry et al. [107] analyzed the miRNA profiles of AH samples from 12 patients with POAG and 12 patients with exfoliation glaucoma (XFG) and compared them to 11 non-glaucoma controls. They identified 3 miRNAs between POAG and controls (miR-125b-5p, miR-302d-3p and miR-451a) and 5 miRNAs (miR-122-5p, miR-3144-3p, miR-320a, miR-320e and miR-630) significantly different between XFG and controls. Pathway analysis revealed that these miRNAs are involved in potential glaucoma pathways, including focal adhesion, tight junctions, and TGF-ß signaling [107]. Some POAG and XFG are associated with vascular dysfunction. Hence, circulating molecules (in plasma) may represent particularly useful biomarkers. Hindle et al. [108] created a customized miRNA-screen to assess expression levels of glaucoma-specific miRNAs in both plasma and AH and to identify candidate biomarkers in glaucoma/XFS patients and cataract controls. They found that circulating levels of 20 miRNAs were higher in glaucoma/XFS patients than in cataract patients, and one miRNA combination (miR-637, miR-1306-5p, miR-3159) demonstrated the best correlation with glaucoma/XFS. Molecular target prediction and pathway analysis revealed potential mechanisms contributing to the pathophysiology of glaucoma, including neuroinflammation signaling, nitric oxide signaling (NOS) and neurotrophin/TRK receptor signaling [108]. 

miRNAs are linked with maintaining the balance of the AH, the change in the TM and the apoptosis of RGCs. Studies on miRNAs in the AH revealed their tissue specificity (17 types of miRNAs may exist only in the AH) [109] and differential expression in glaucoma patients (11 types of miRNAs were significantly upregulated and 18 types of miRNAs were significantly downregulated) [110]. miRNAs can also influence changes in the TM by regulating gene expression in its tissue [111]. A complex regulatory system in the TM tissue could affect the occurrence and development of glaucoma by influencing the extracellular matrix and the contraction as well as the aging of its cells [112]. miRNA could control TGF-β which affects the metabolism of extracellular matrix in the TM. Overexpression of miRNA-24 was shown to downregulate the expression of TGF-β [113]. The miRNA-29 family might regulate the effect of TGF-β on the extracellular matrix [114]. Several studies found that miRNA-483-3p had an inhibitory effect on extracellular matrix production in the human TM cells through downregulating Smad4, which targeted TGF-β/bone morphogenetic protein and laminin [115,116]. These observations turn miRNAs into potential therapeutic targets in influencing the development of glaucoma by regulating TGF-β which affects the extracellular matrix.

Oxidative stress plays an important role in the aging of TM cells. The expression of miRNA in TM is changed under the condition of oxidative stress, as shown by Li et al. [117] who found that 14 types of miRNAs were downregulated and 3 were upregulated in the TM cells after the treatment with hydrogen peroxide (H_2_O_2_)_._ Other studies of the same group of authors found that miRNA-204 and miRNA-183 may regulate the apoptosis of the TM, which influences the IOP [117,118]. 

The apoptosis of RGCs is the main cause of optic nerve damage in glaucoma, therefore understanding the mechanisms of RGCs apoptosis is very important for treating glaucoma. miRNAs are expressed in the retina of eyes with advanced glaucomatous damage [119]. Upregulation of miRNA-96 could decrease the activity of RGCs, through the activation of caspase-2 [120]. Conversely, downregulation of miRNA-100 reduces the apoptosis of RGCs and promotes nerve growth through the phosphorylation pathway [121]. 

In a study regarding the potential therapeutic benefits of MSCs, intravitreal infusion of MSCs promoted RGCs survival in a mouse model of acute glaucoma [122]. The mechanism of this MSC-mediated neuroprotection involves the miRNA-21 and its target PDCD4 who regulate MSCs to secrete mainly STC1 (Stanniocalcin 1), but also other neuroprotective factors. Therefore, modulation of the miRNA-21/PDCD4 axis represents a promising tool for improving the neuroprotective and anti-inflammatory effects of MSCs. 

In the light of the above, miRNAs represent a hotspot in medicine, particularly in ophthalmology. It is already known that they are associated with many ocular diseases, but studies in glaucoma are still in the primary stage. However, the results of these studies are very promising and validation of the specific expression of miRNAs in glaucoma should become the focus of future research due to their potential clinical applications: biomarkers for early diagnosis, prognosis and outcome, as well as development of novel therapeutic strategies in glaucoma.

## 5. Conclusions

Surprisingly, there are few clinical trials using stem cells and their derivates in glaucoma, and most have not reported any results, although animal studies have been very promising. It can be assumed either that the approach in these trials was not the most appropriate, or that the glaucomatous optic nerve atrophy was so advanced at the time of diagnosis that it could no longer regenerate and the neural circuits could no longer recover, even in the presence of neurotrophic factors. Another possible explanation is that in animal studies the lesions were produced in a short period of time and were not irreparable, while glaucomatous disease in humans evolves insidiously, over years, and leads to complex and irreversible neuronal damage.

Autologous stem cells are the most indicated in transplantology. Pluripotent cells, such as iPSC and ESCs, are more difficult to obtain. Satisfactory results with MSCs appear to be obtained if they are transplanted at the right time and place and if carriers for growth factors and bioactive molecules, such as immunomodulators and neurotrophic factors, are taken into account. It is important to remember that standardization of stem cells and exosomes harvesting, cultivation and characterization (phenotipic, genetic and especially functional) is necessary for clinical trials to be successful and reproducible.

The most favorable route of administration in glaucoma, in terms of safety and risk of immune response, seems to be the suprachoroidal space. 

A viable and promising alternative is the use of exosomes and miRNAs that would reduce the risks of unpredictable stem cell transplants.

## Figures and Tables

**Table 1:** Clinical trials investigating stem cell therapies in glaucoma and optic nerve atrophy that are registered at the National Institute of Health.

Study	ClinicalTrials.gov Identifier	Disease Target	Therapy	No of Subjects Enrolled	Results/Publications
Stem Cell Ophthalmology Treatment Study (SCOTS1) 2013–2019 USA	NCT01920867	AMD, RO, Glaucoma	Autologous bone marrow derived stem cells	300	[69]
Stem Cell Ophthalmology Treatment Study (SCOTS2) 2017–2021 USA	NCT03011541	AMD, RO, Glaucoma	Autologous bone marrow derived stem cells	500	No results reported
Effectiveness and Safety of Adipose-Derived Regenerative Cells for Treatment of Glaucomatous Neurodegeneration 2014–2019, Russian Federation	NCT 02144103	Retinal degeneration, POAG	Autologous adipose-derived regenerative cells	16	No results reported
Study the Safety and Efficacy of Bone Marrow Derived Autologous Cells for the Treatment of Optic Nerve Disease (OND) 2014–2016, India	NCT 01834079	Optic nerve disease	Bone marrow derived autologous mononuclear cells	24	No results reported
Intravitreal Mesenchymal Stem Cell Transplantation in Advanced Glaucoma, 2015–2019, Brazil	NCT02330978	Advanced glaucoma	Bone marrow derived stem cells	2	[67]
Cord Blood Serum in the treatment of Neuro- Degenerative Ophthalmic Diseases. 1-Glaucoma 2018, Italy	NCT03609125	Glaucoma	Cord blood serum eye drops in glaucoma patients	10	No results reported
NT-501 CNTF Implant for Glaucoma: Safety, Neuroprotection and Neuroenhancement (2011–2016) USA	NCT01408472	Glaucoma	NT-501 CNTF implant (made by Neurotech)	11	No results reported
Treatment of Optic Neuropathies using Autologous Bone Marrow-Derived Stem Cells 2015–2021, Arabia	NCT02638714	Optic Nerve Atrophy	Autologous purified populations BM-SCs	100	No results reported
Safety Assessment of Intravitreal MSCs for Acute Non Arteritic Anterior Ischemic Optic Neuropathy (NEUROSTEM) 2017–2021, Spain	NCT04877067	NAION	Allogenic MSCs	5	No results reported
Therapy of Toxic Optic Neuropathy via combination of stem cells with Electromagnetic Stimulation (Magnovision) 2021, Turkey	NCT04877067	Toxic Optic Neuropathy	Wharton’s jelly-derived MCSs in sub-tenon space + repetitive EMS	18	[74]
